# Supplementary material for: 3D chromatin-based variant-to-gene maps across 57 human cell types reveal the cellular and genetic architecture of autoimmune disease susceptibility
Source: Genome Biol. 2025 Dec 8;26:414. doi: 10.1186/s13059-025-03880-4 (PMC12683900; doi:10.1186/s13059-025-03880-4)

Figure S1. Full S-LDSC parameters across diverse cell types' cREs annotation. A. Bar plots (left) is the same in Figure 1A. The dot plots depict heritability enrichment for each cell type across 16 autoimmune traits as determined by LDSC analysis. Whiskers represent enrichment standard errors, with colors matched for HiC vs. capture-C. The colors of the dots correspond to p-values in -log10, with dots featuring a white asterisk indicating a significant p-value ≤ 0.05. The size of the dots corresponds to the proportion of SNP contribution to heritability. A dashed line at 1 indicates no enrichment. B. Bar plots (left) is the same in Figure 1A. The bar plot depicts conditional effect sizes for each cell type's cREs annotation across 16 autoimmune traits as determined by LDSC analysis. The gradient color of the bar corresponds to the p-value in -log10, with a framed bar indicating a significant p-value ≤ 0.05. Bars with red asterisks passed FDR ≤ 0.05.

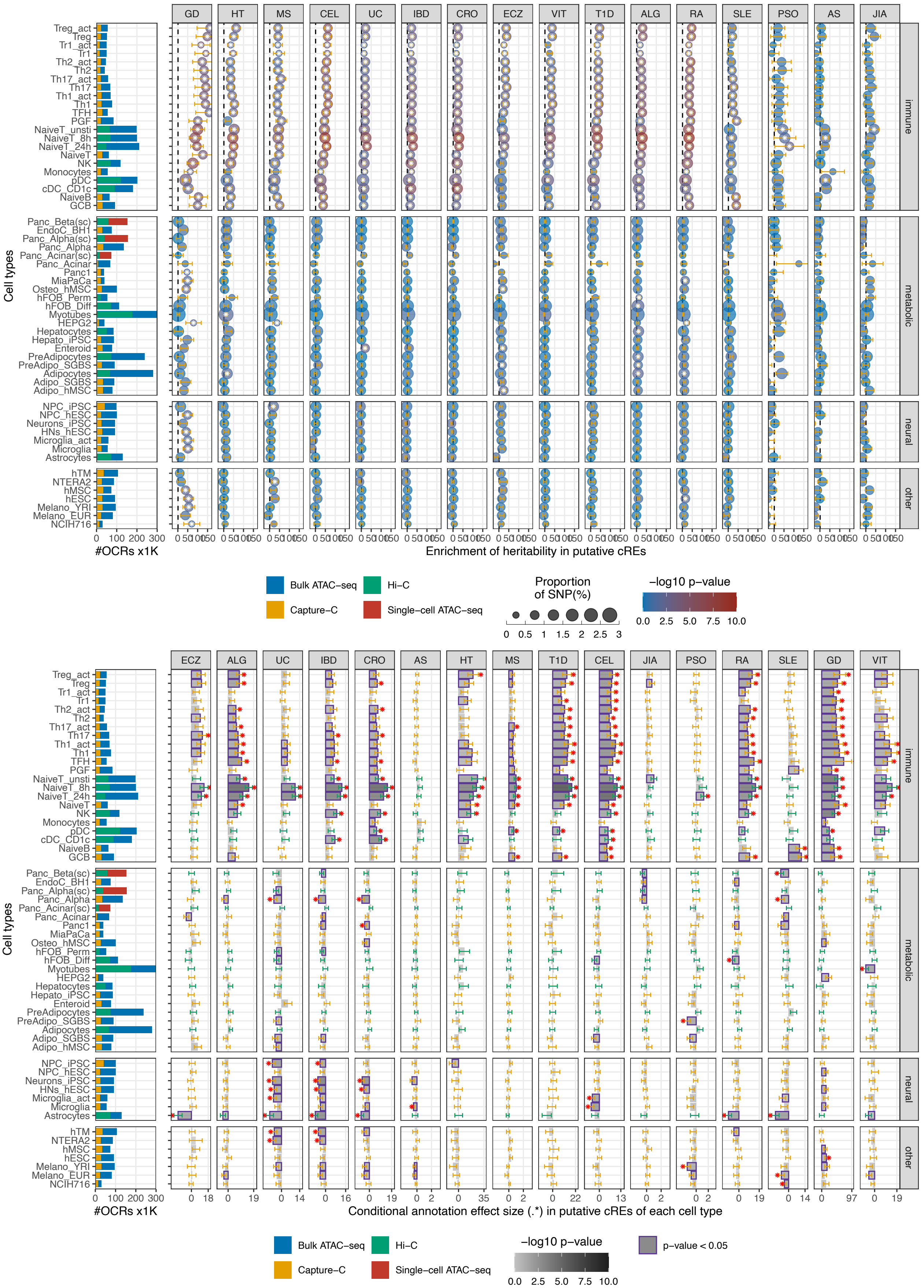

Figure S2 - Plots show number of significant (P<0.05) heritability enrichment for cell types per trait (A) and for traits per cell type (B).

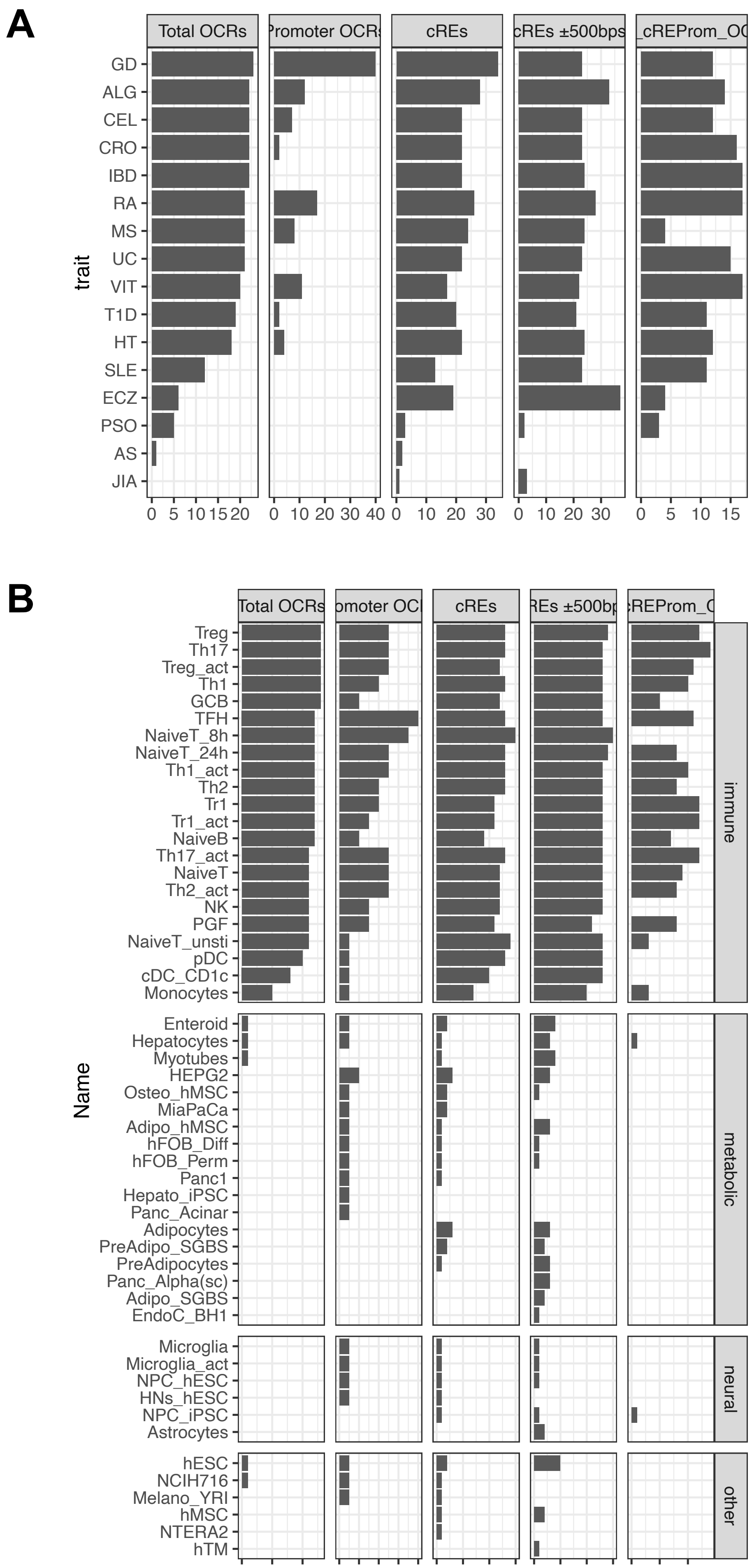

Figure S3 - Bar plots show number of significant (P-values < 0.05) conditional effect sizes (whole bars) and FDR<0.05 (green portions) for cell types per trait (A) and for traits per cell type (B). Volcano plots show conditional effect sizes versus P-values for cell types per trait (C) and for traits per cell type (D), red dashed line is threshold p-value=0.05.

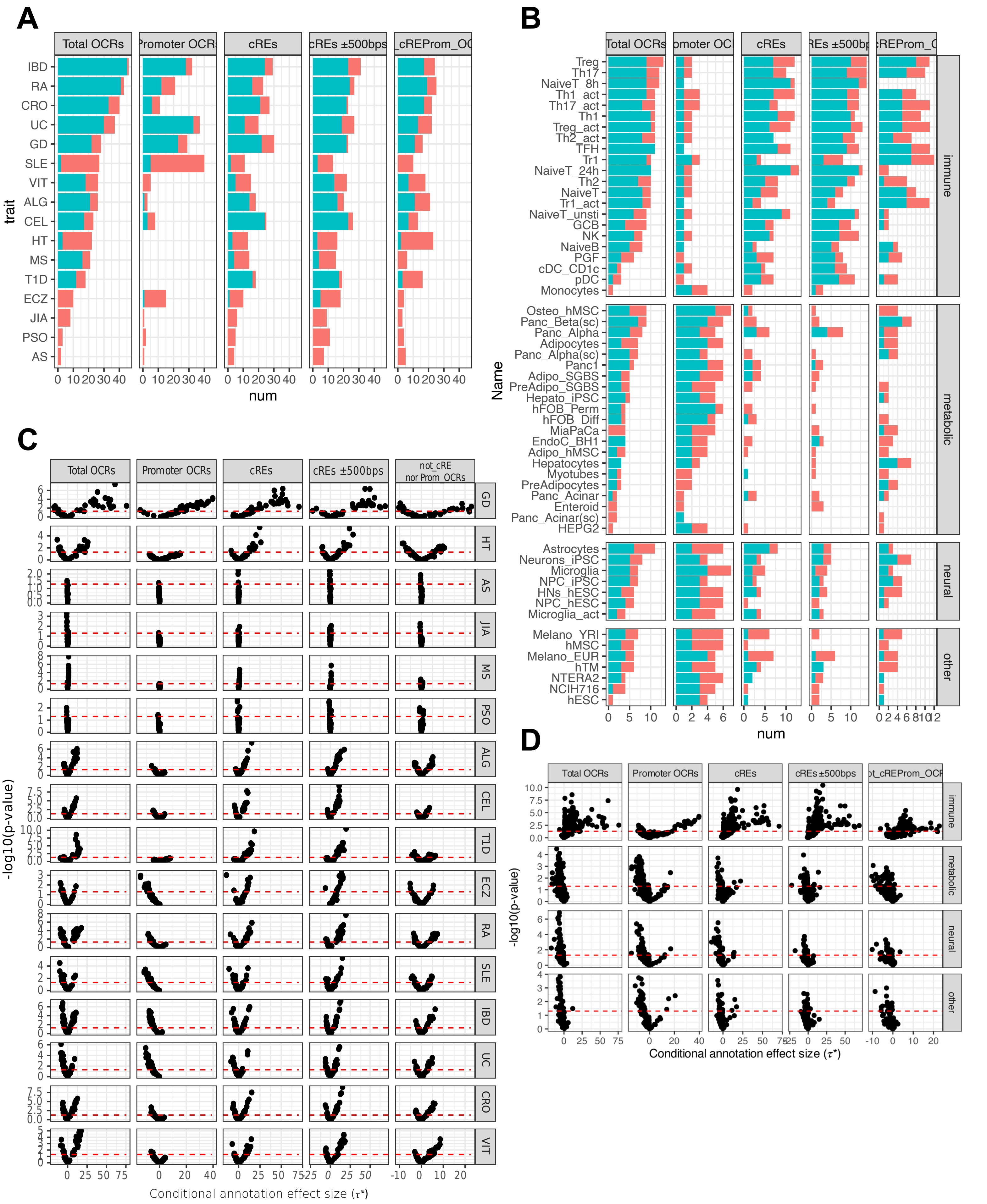

Figure S4 - (A) Upset-plot showing intersections of V2G genes implicated in any cell type across 16 immune traits. (B) Upset-plot depicting intersections of V2G genes for any trait across the cell types listed. (C) Degree of overlap of implicated variants and target genes (D) across cell types

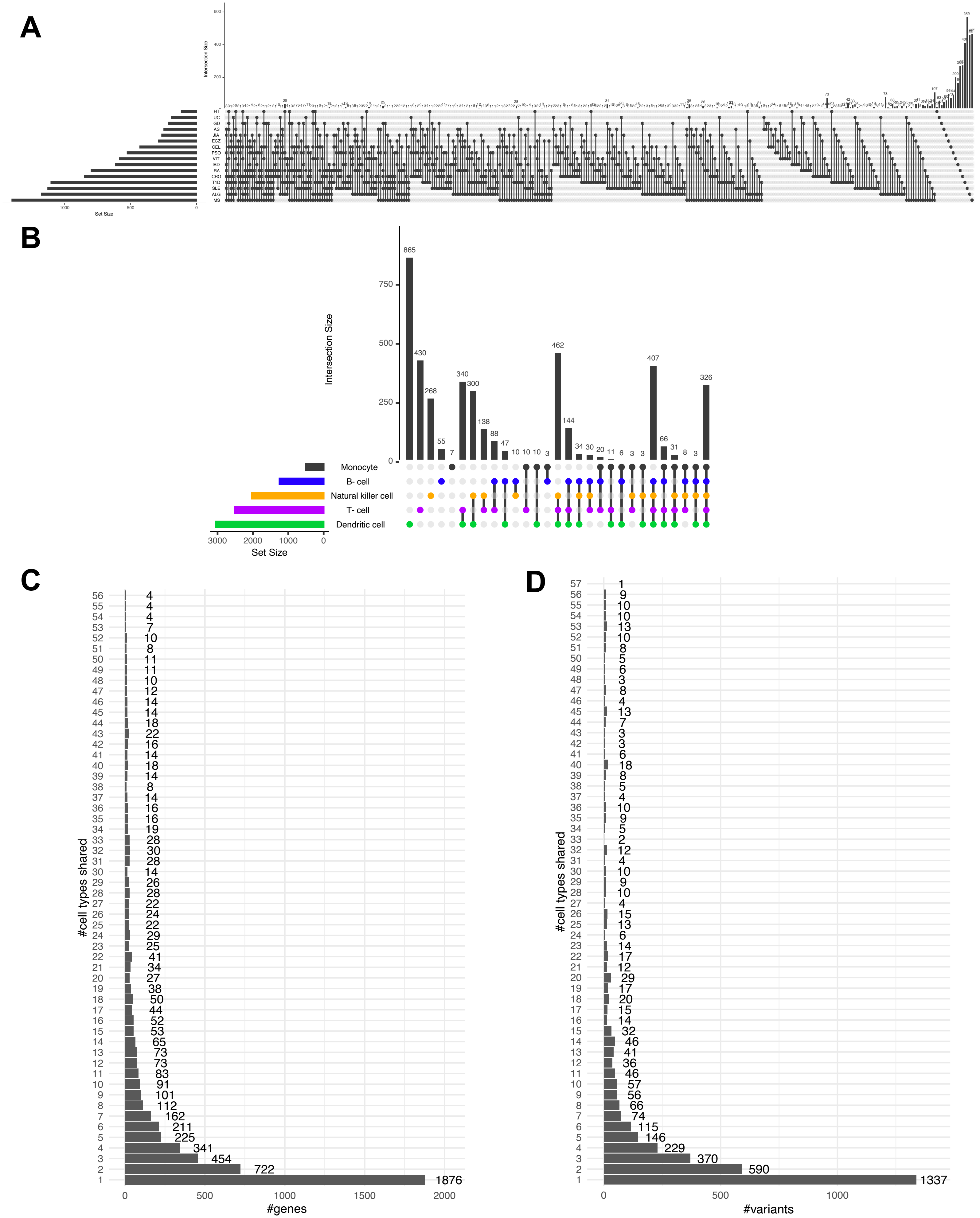

Figure S5 - Comparative predictive power of orthogonal variant-to-gene (V2G) approaches. (A) VennDiagrams showing overlaps in predicted genes between our immune cell data versus other chromatin-based V2G approaches. (B) Precision-recall analysis of V2G gene predictions against the set of monogenic human inborn errors in immunity.

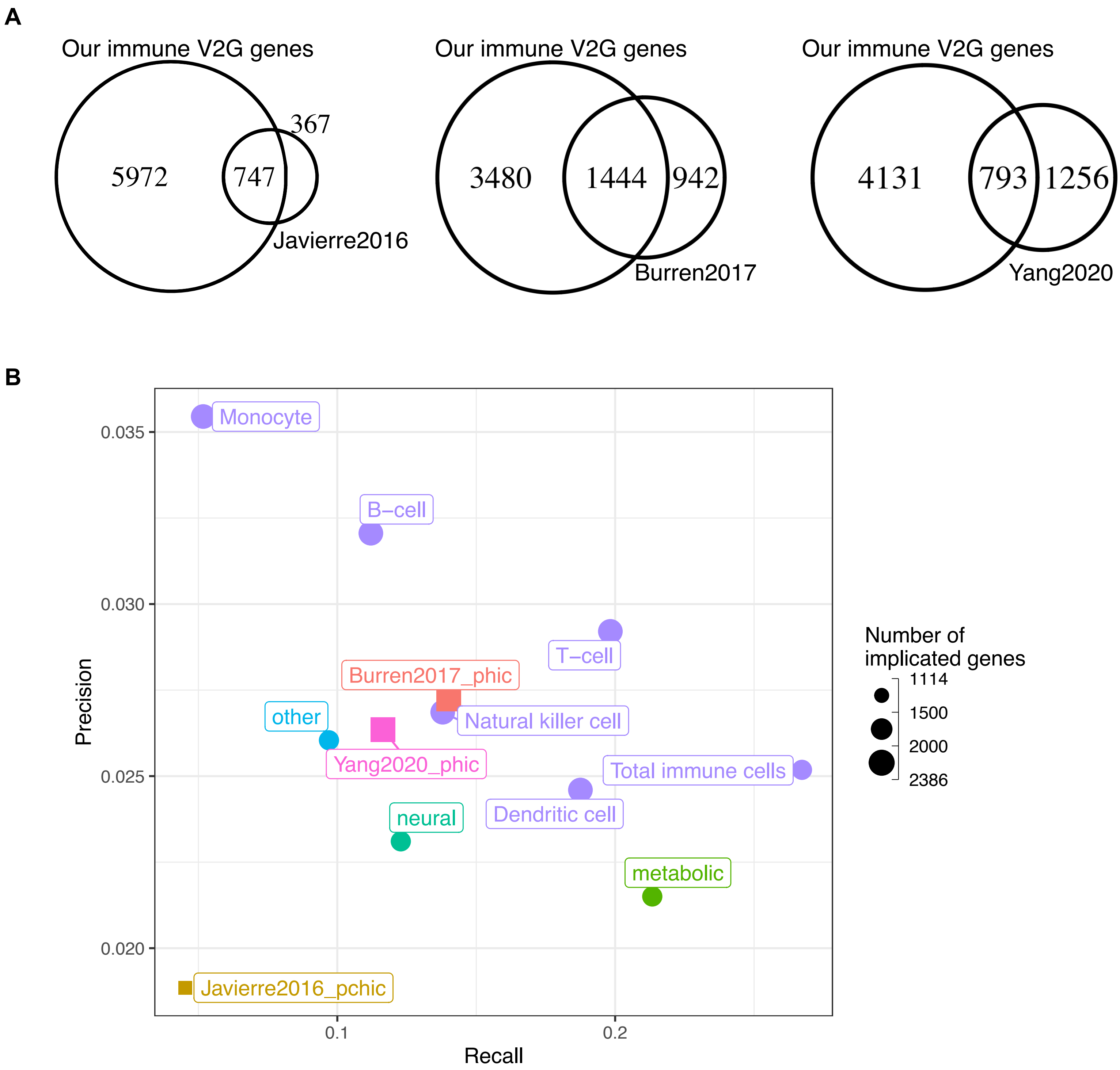

Figure S6 - Cytokine/receptor gene enrichment across trait and cell type (A) and in KEGG pathway (B)

A

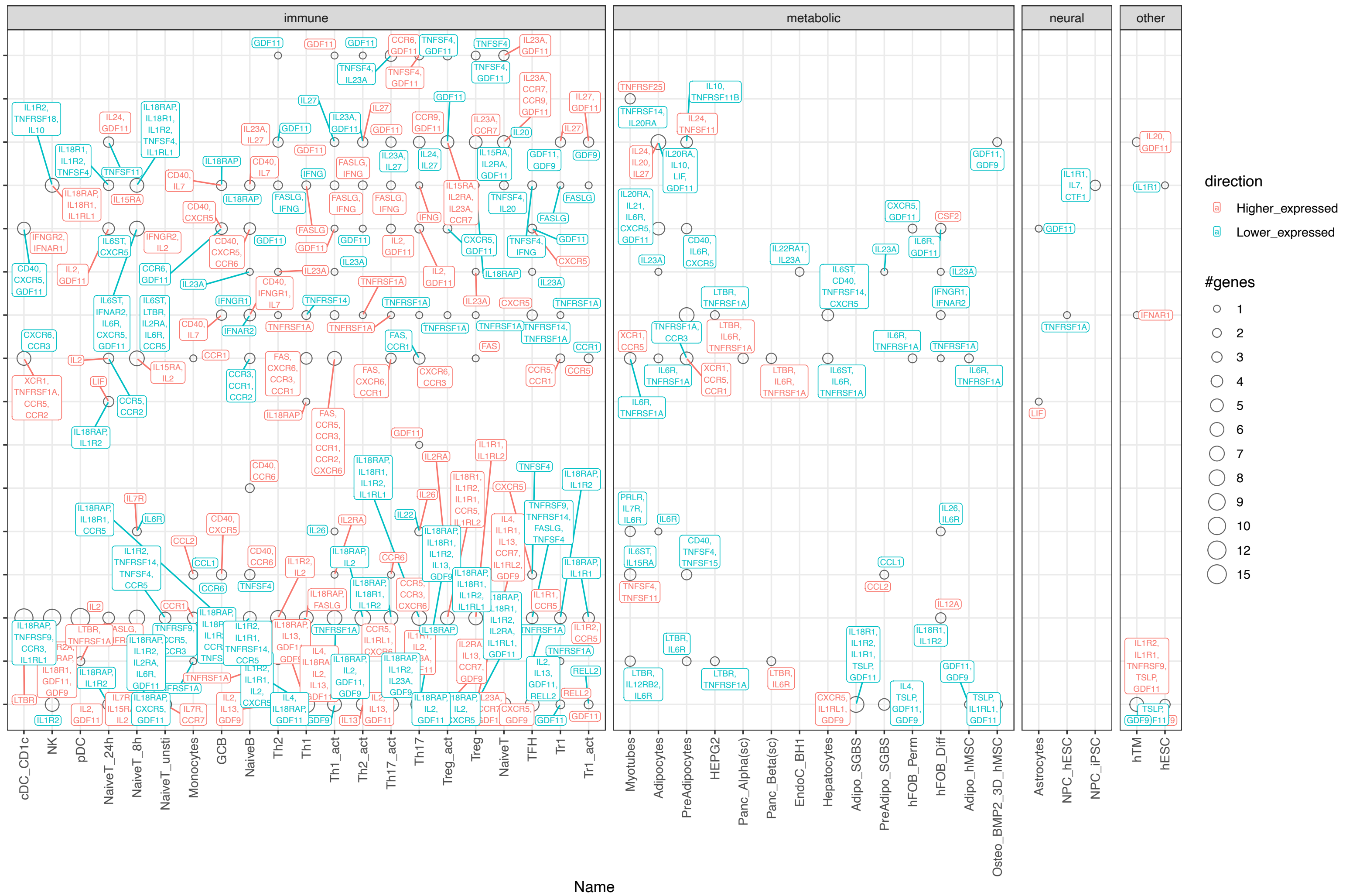

B

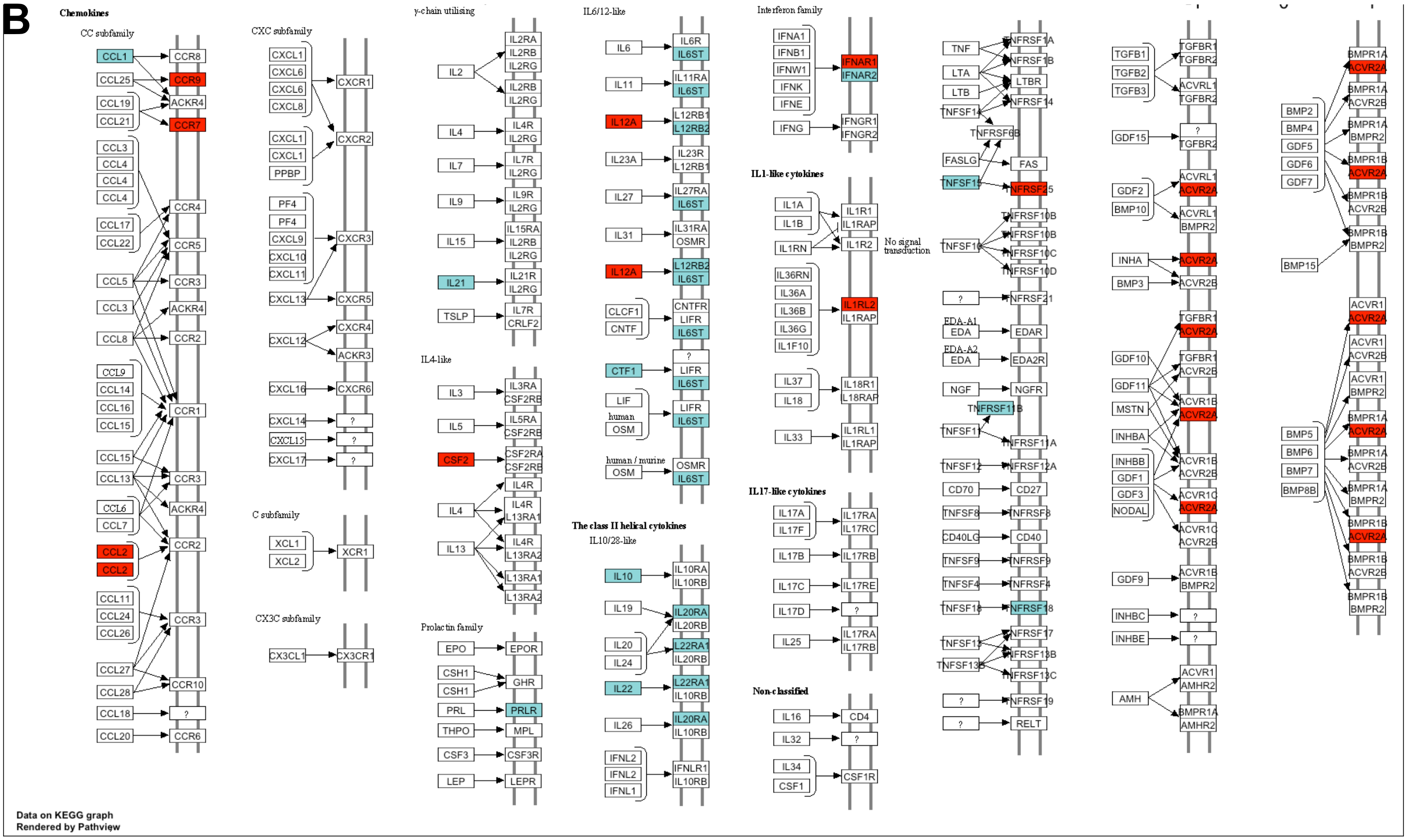

Figure S7 - Salmonella infection gene enrichment across trait and cell type (A) and in KEGG pathway (B).

A

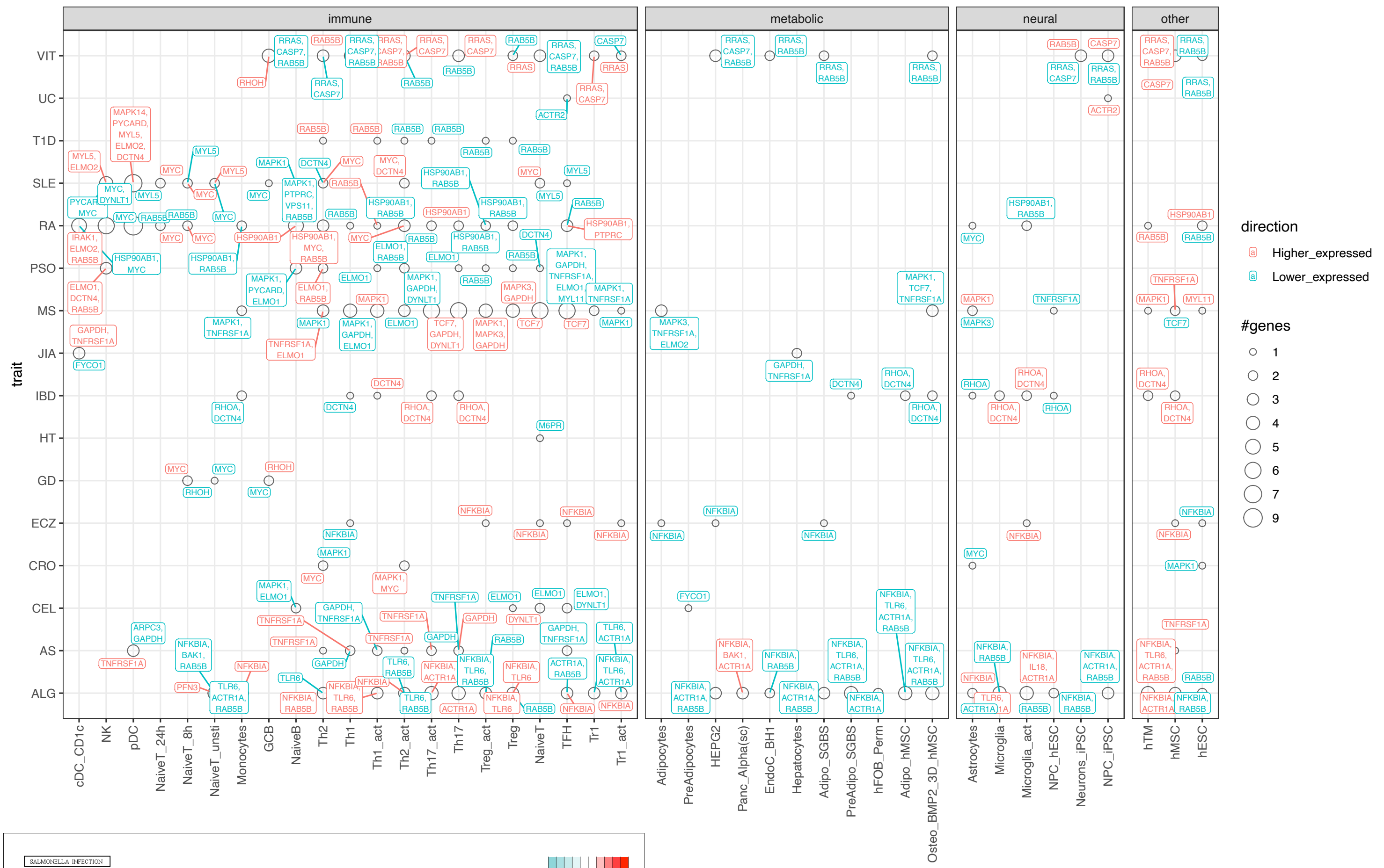

B

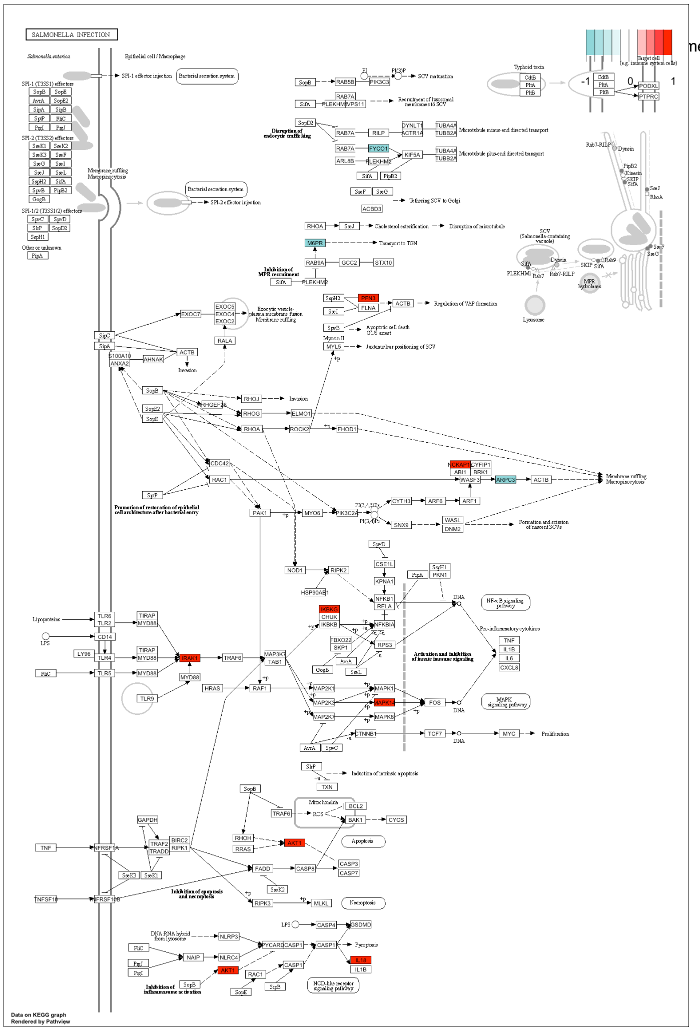

Figure S8 - Sharing of V2G genes in enteroids across UC, CRO, and IBD (A). Gene ontology (B) and pathway enrichment (C) across each trait are shown.

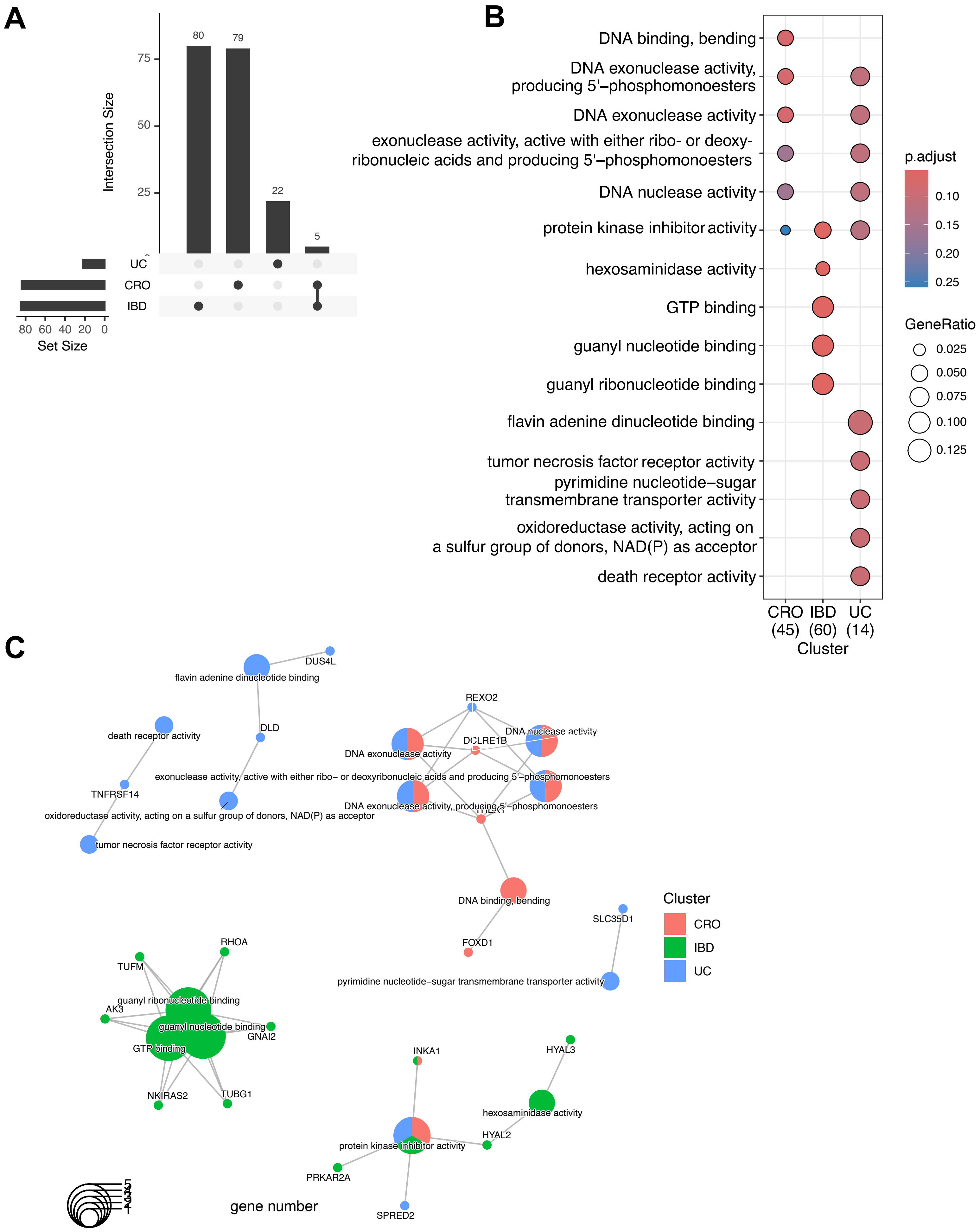

Figure S9 - Gene ontology enrichment of cell type-specific V2G genes across cell type.

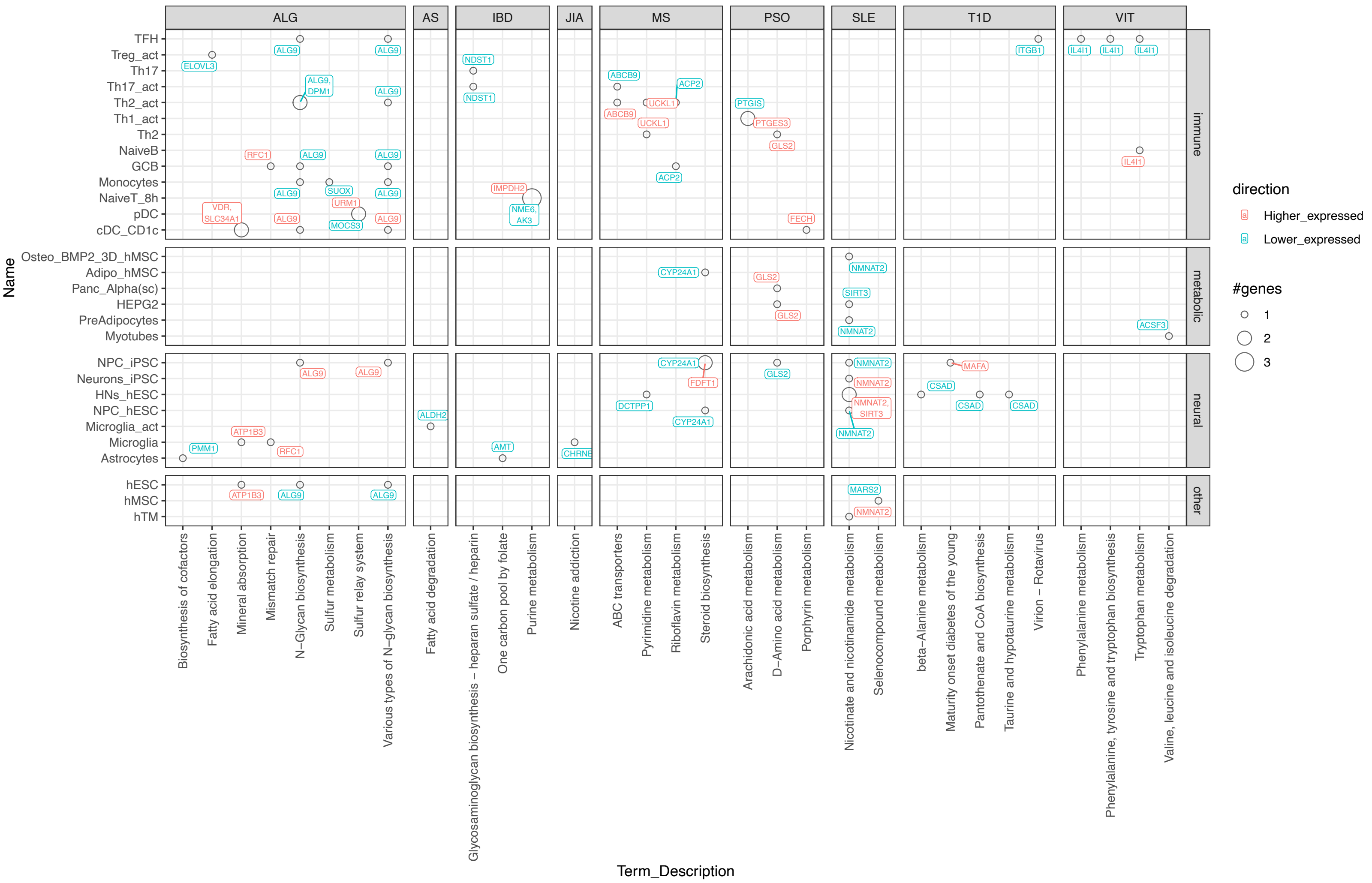

Figure S10 - Shared eGenes across different eQTL datasets.

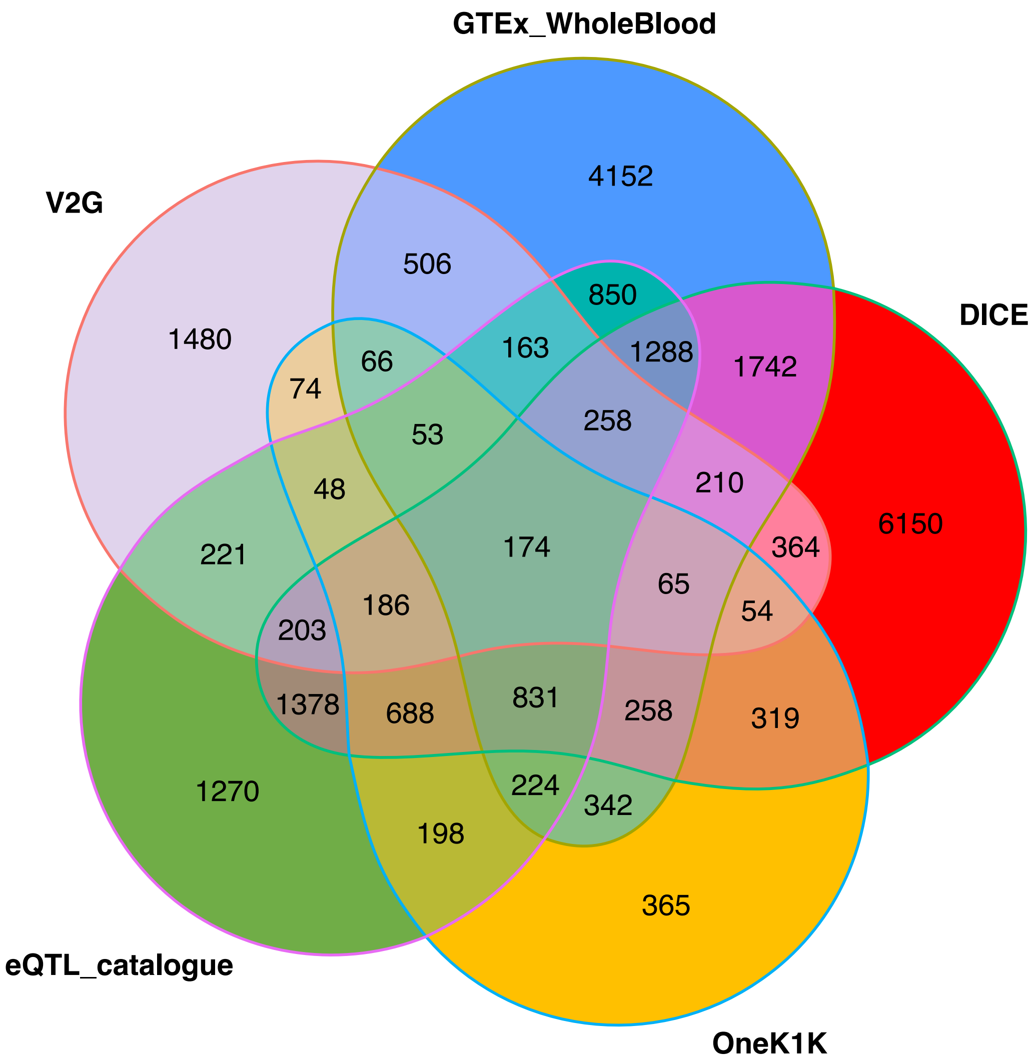

Figure S11 - Shared eGenes across different eQTL datasets with V2G

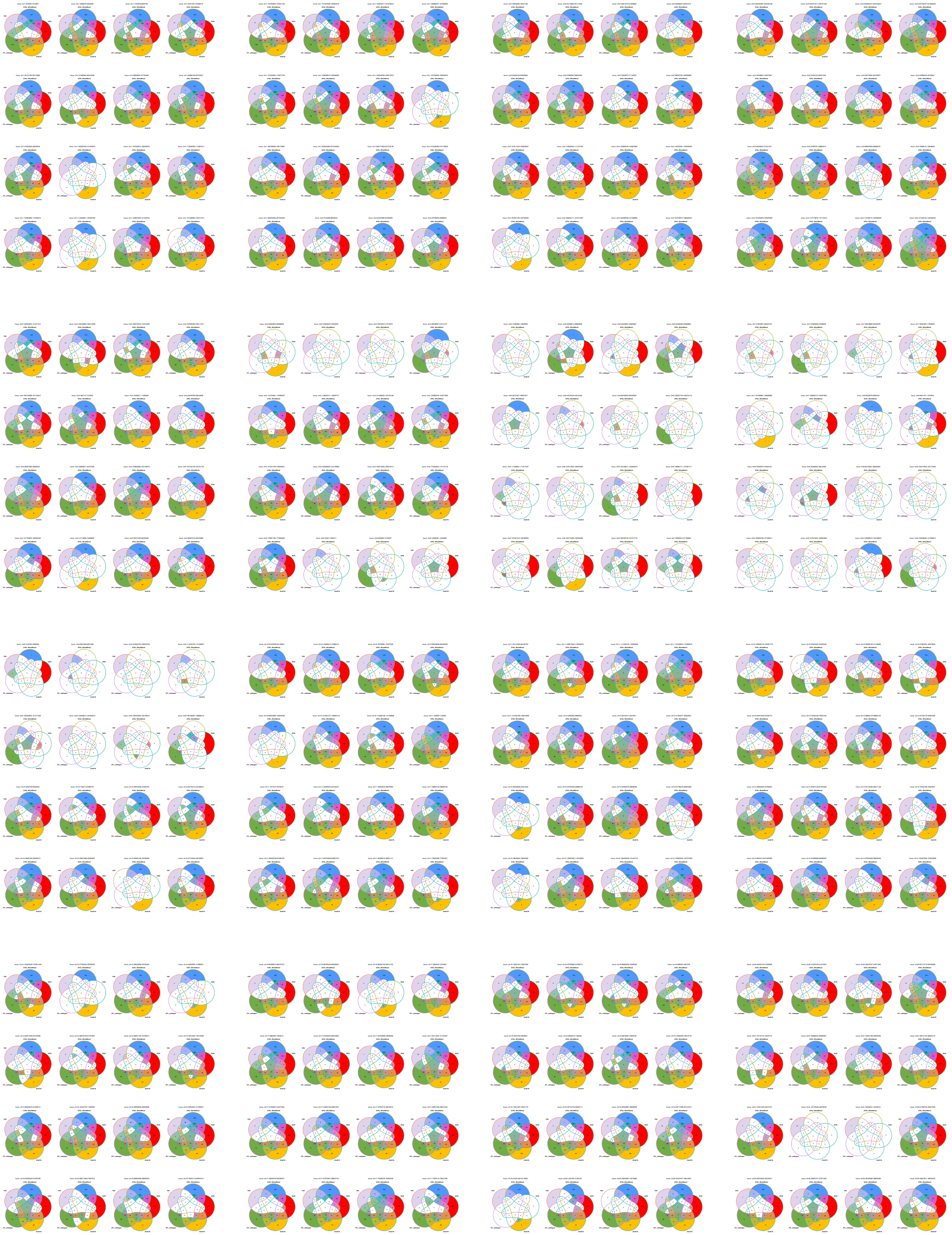

Figure S12 - Collage of pie plots each depicting the proportion of eGenes identified by eQTL that match (light orange) or do not match (purple) the gene identified by 3D chromatin V2G for each cell type-trait pairing. Left panels: bar plots depicting the total number of eQTL eGenes for each immune cell type that match or differ across all autoimmune traits. Bottom panels: bar plots depicting the total number of eQTL eGenes for each trait that match or differ across all immune cell types.

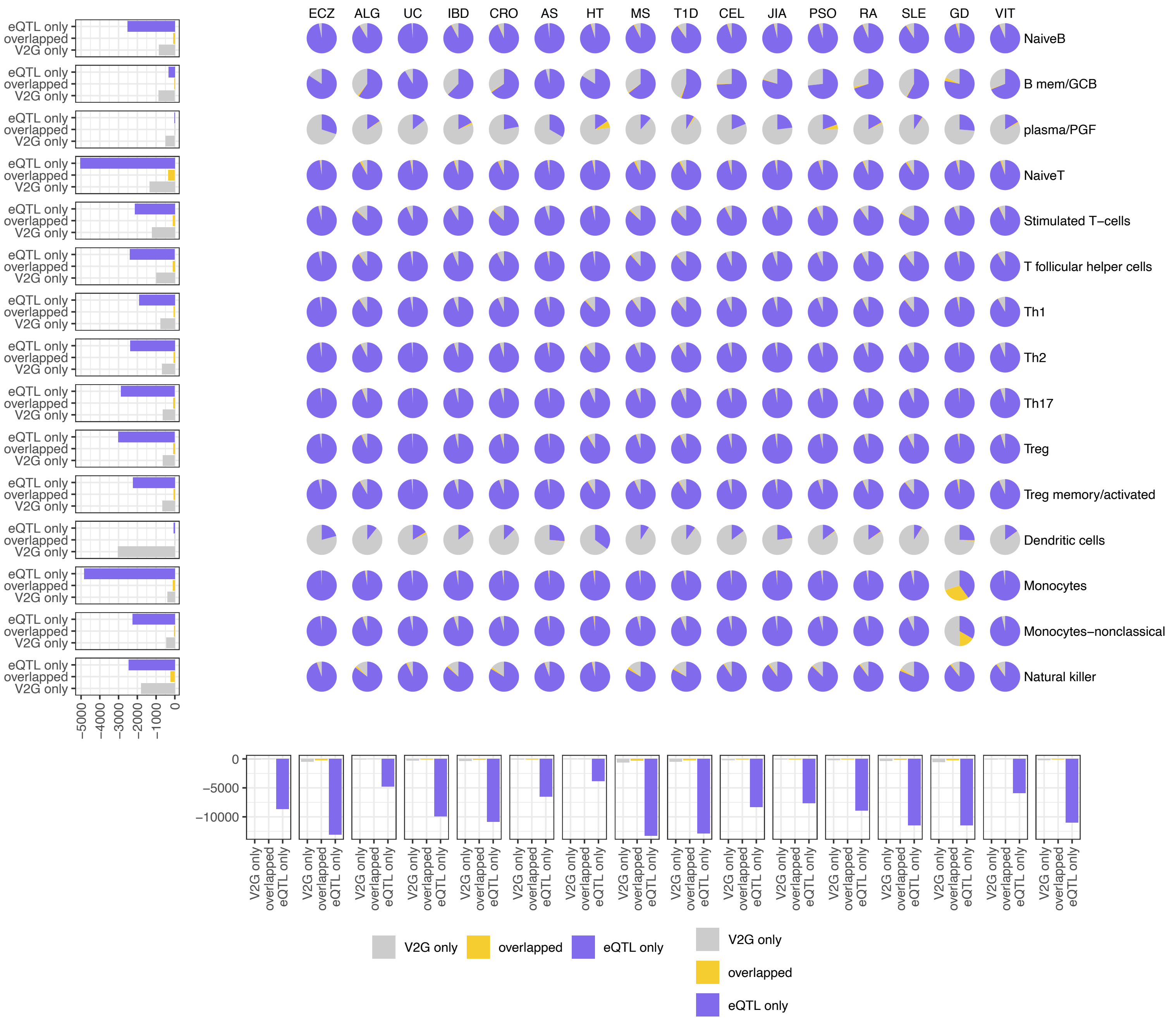

Figure S13 - Disrupted transcription factor binding motifs: Motifs of transcription factors where binding sites were predicted to be affected by high-probability causal variants (SNPs with PP.H4.abf ≥ 0.8) that overlapped with variants identified by the V2G (variant-to-gene) approach. Altered alleles are presented, consistent with the corresponding GWAS of the immune traits.

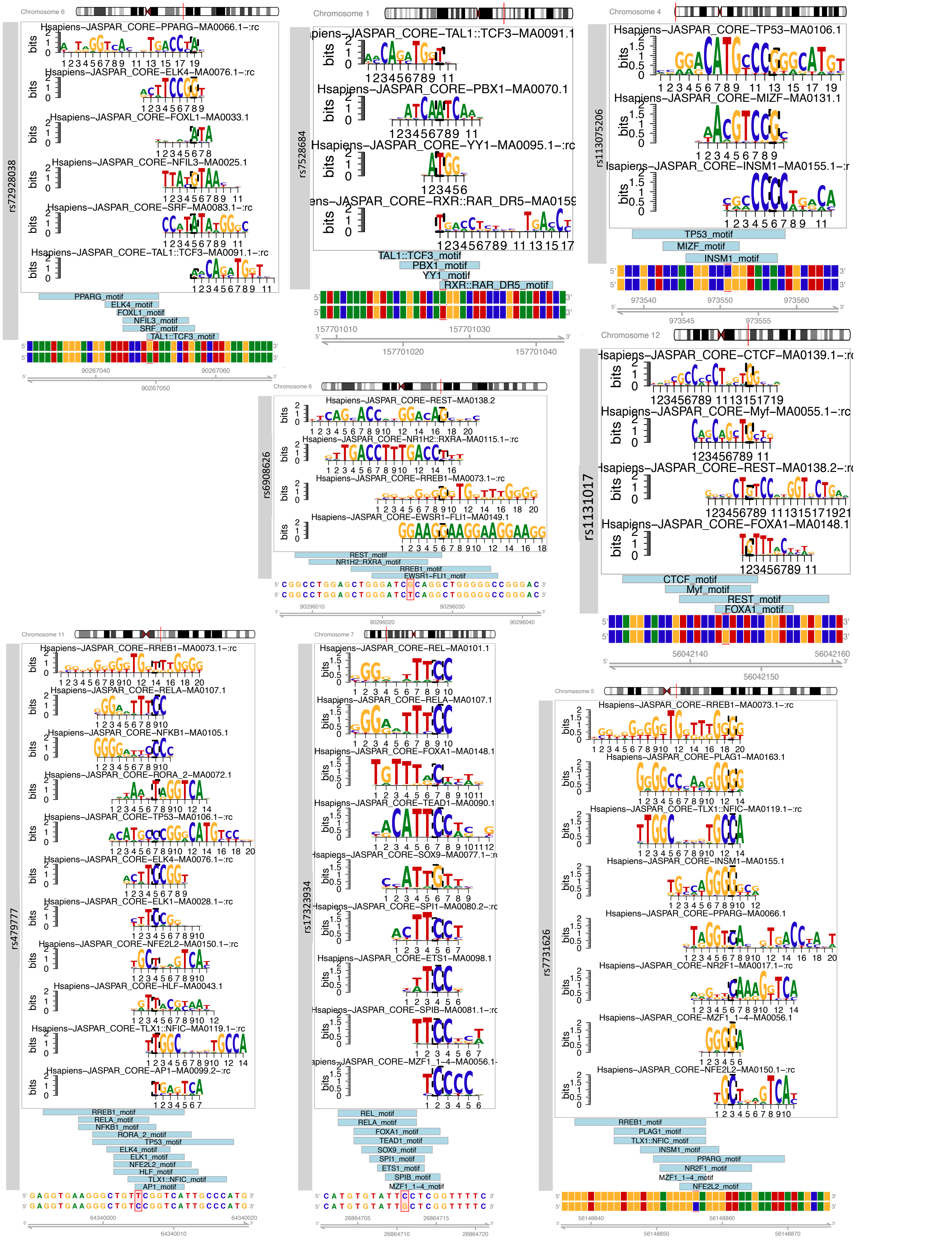

Figure S14 - Dot-plot shows effect sizes of SLE and RA variants on FDFT1 expression in different cell types, shaped according to the origin of datasets, colored by whether the variant were SLE leads or their proxies.

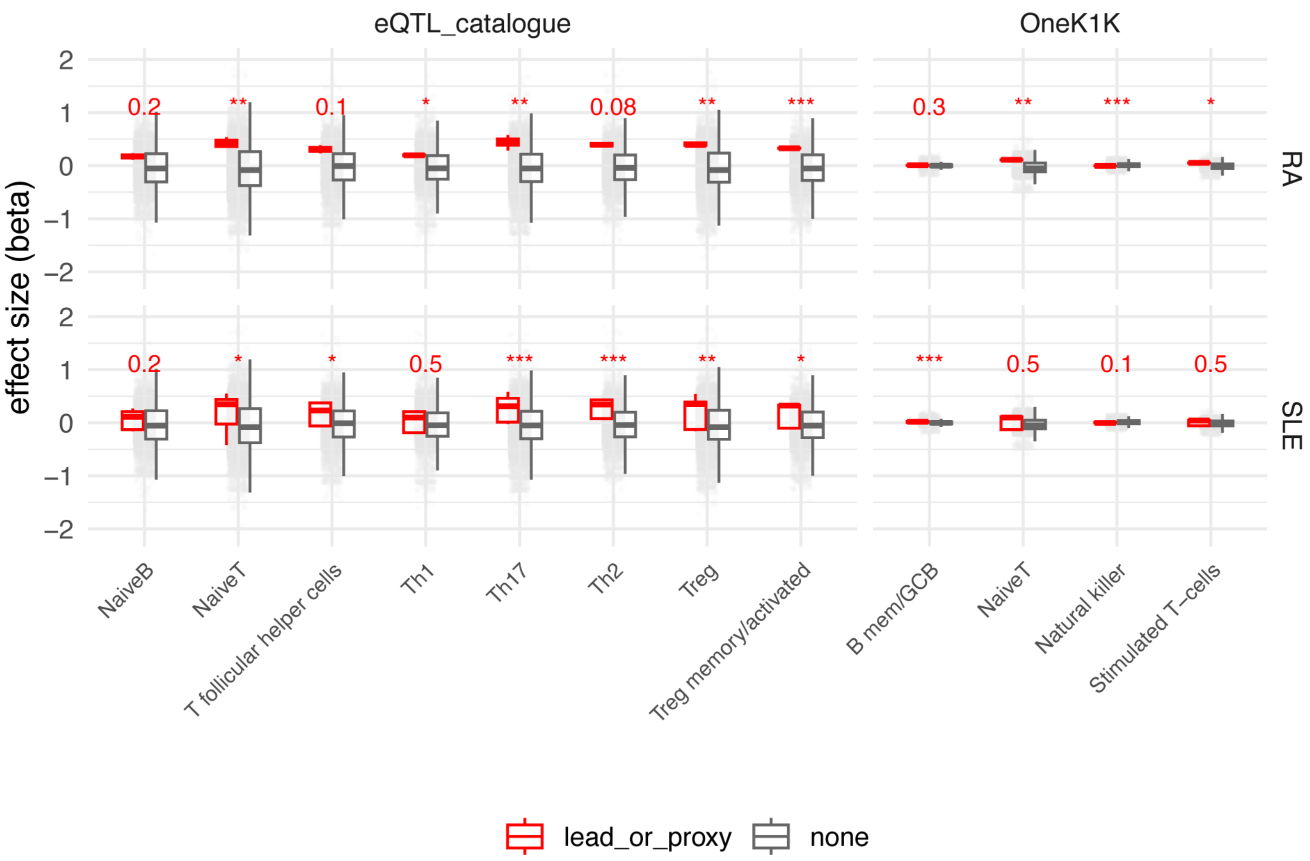

Figure S15 - Expression of BLK in sorted immune cells from RA patients, SLE patients, and healthy subjects measured by bulk RNA-seq. Statistically significant differential expression compared to healthy subjects is denoted by p-values.

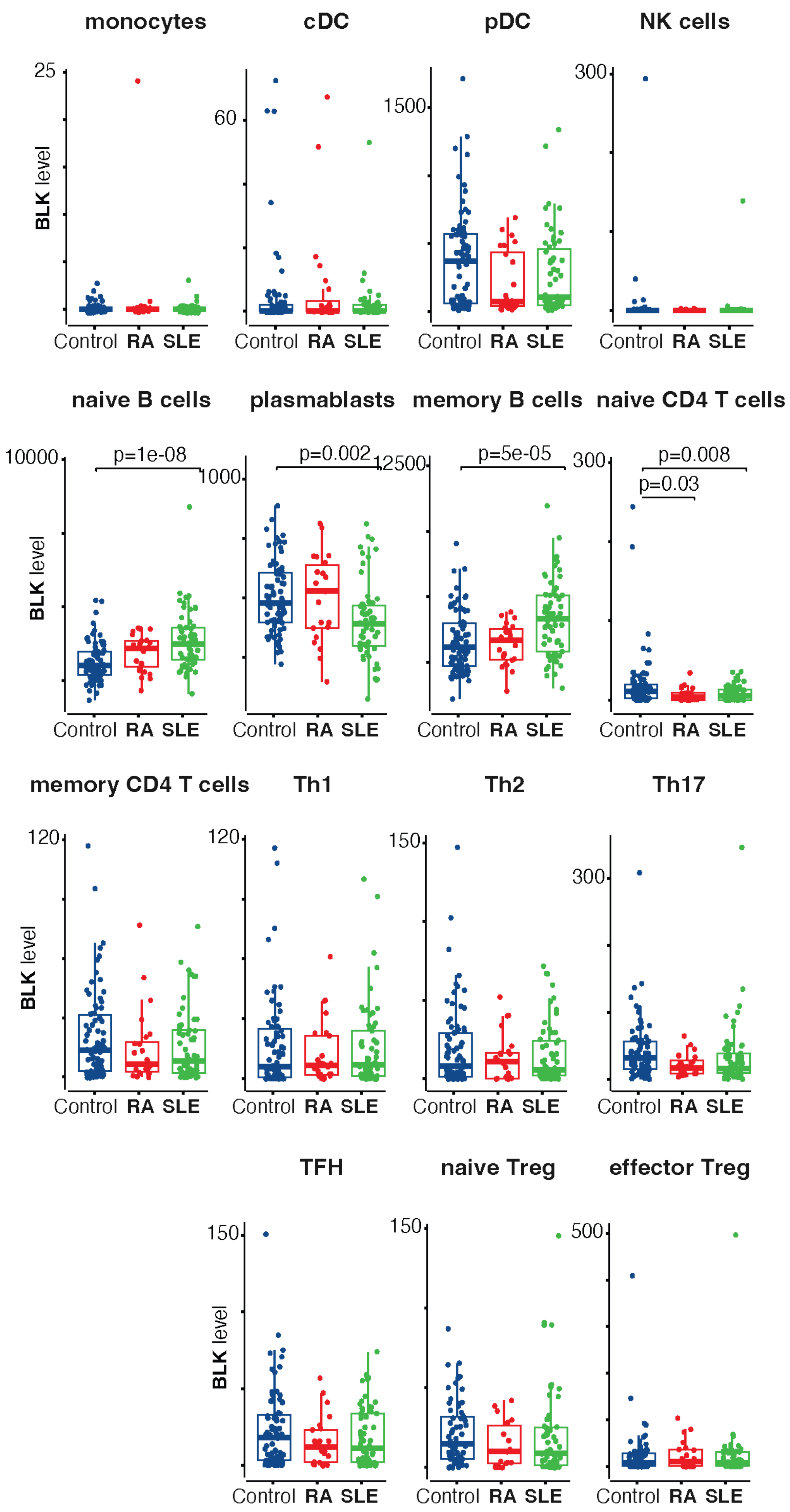

Figure S16 - Effect of lapaquistat on T cell activation as measured by induction of IL-2 receptor (A) and IL-2 (B) expression and proliferation (C) by human CD4 T cells stimulated with anti-CD3+CD28 beads.

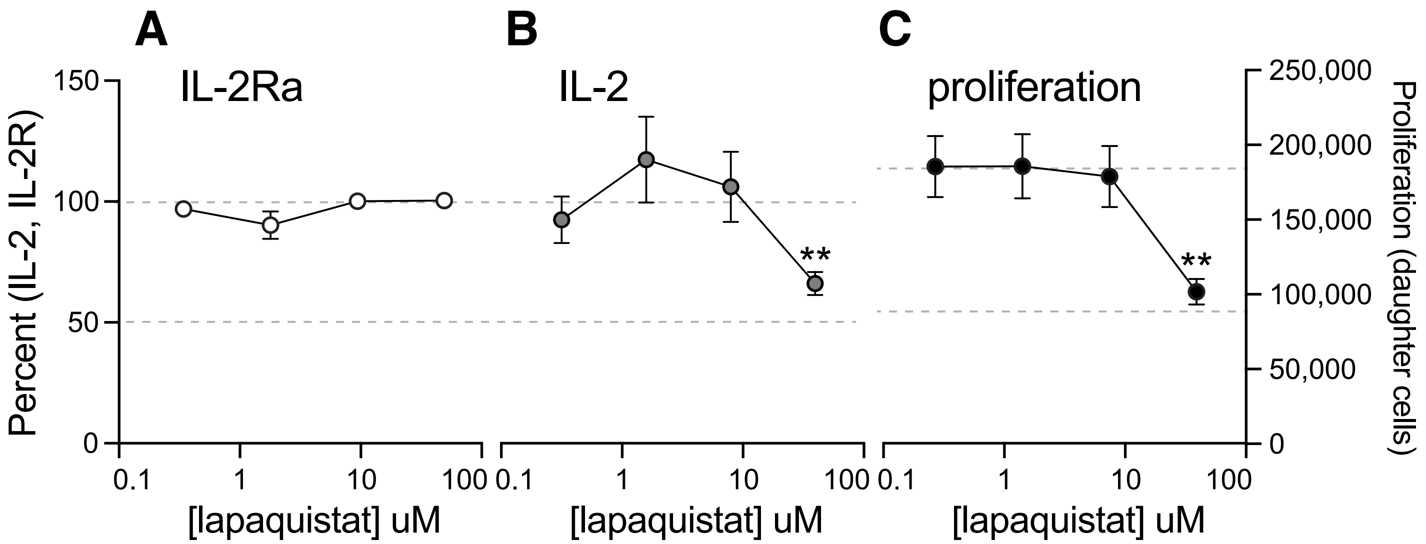

Figure S17 - Gating Strategy

(A) Gating strategy for sorting of plasmacytoid dendritic cells (pDC) and CD1c+ dendritic cells (cDC2). cells are gated to identify Lymphocytes , which are then refined to select for Single Cells and Live cells (by excluding a live/dead dye). From this population, Lineage- cells are identified , and HLADR+ cells are subsequently gated. This HLADR+ population is then separated into Plasmacytoid DCs and Conventional DCs. Finally, the Conventional DC population is further resolved into CD141+ conventional DCs and CD1c+ conventional DCs.

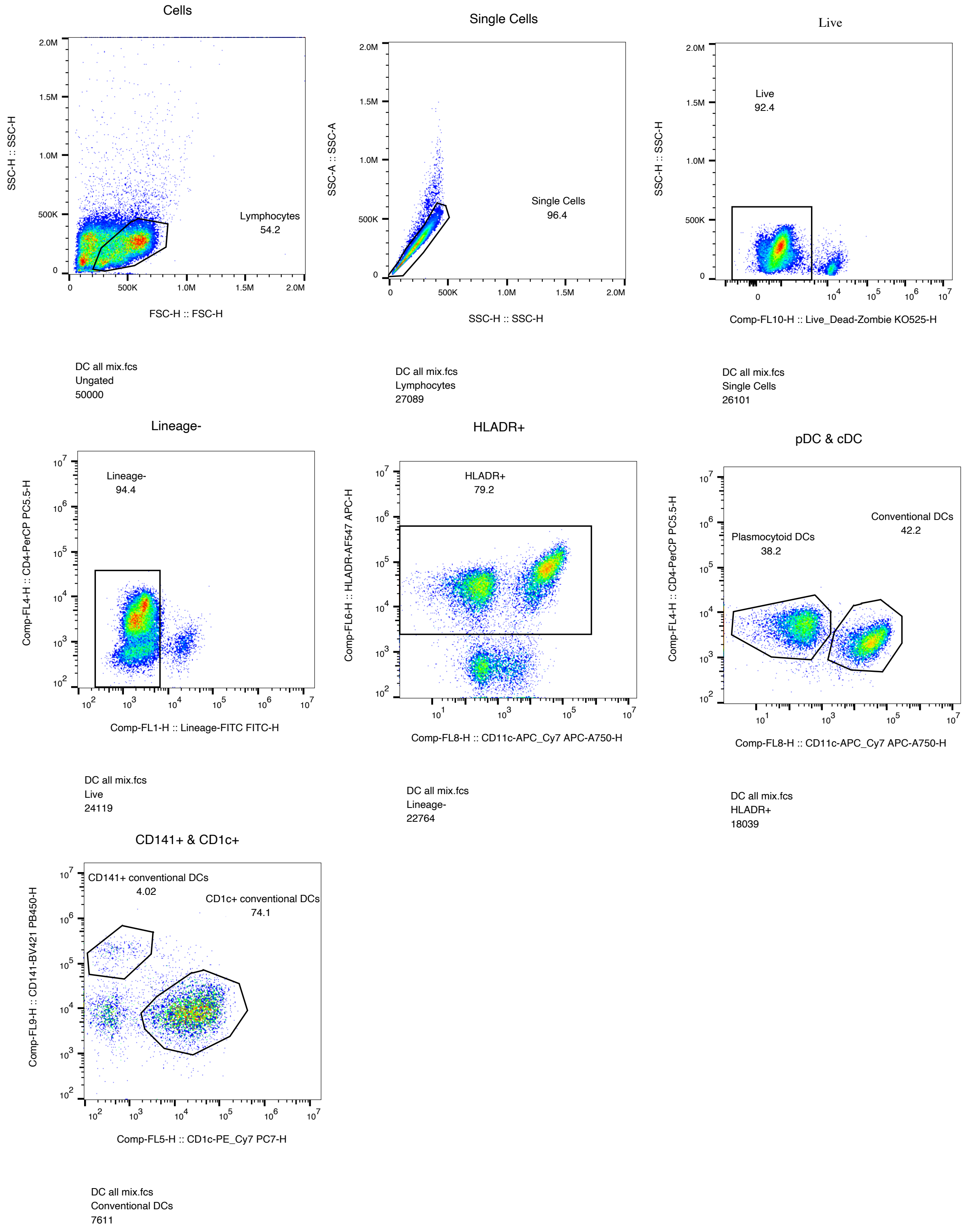

(B) Tregs are identified from CD25-enriched cells as the CD25+ CD127- population. A post-sort plot confirms 97.0% purity. CD25-depleted cells (Tconv) are first gated based on CXCR3 expression. Th1 cells are sorted from the CXCR3+ population and are defined as CCR6- CCR4-.Th2 cells and Th17 cells are sorted from the CXCR3neg population. Th2 cells are defined as CCR6- CCR4+ , and Th17 cells are CCR6+ CCR4+. Purity checks for all three subsets are shown.Tr1 cells are identified from IL10-PE-enriched cells gated as CD4+CD45RAneg and IL10+.

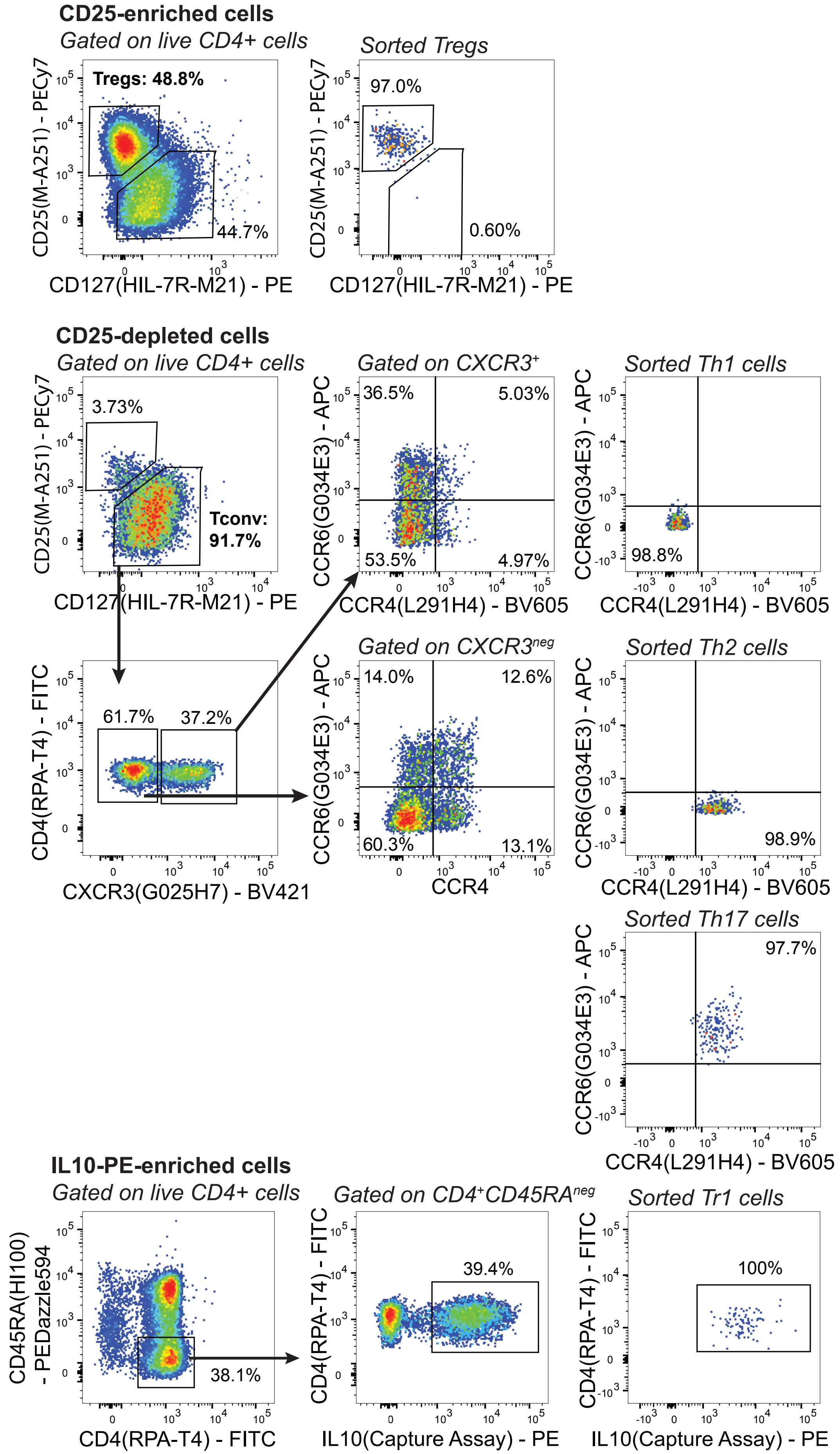

Figure S18 - (A) Phenotypic Characterization of Sorted T Helper Subsets Th1, Th2, Th17, Tr1, and Treg subsets. See Additional File 1: Table S13 for donor info.
Top (Histograms): Expression of the transcription factor T-bet is shown, with Th1 cells having the highest expression. Expression of the transcription factor GATA3 is also shown, with Th2 cells having the highest expression.
Bottom (Flow Plots): Chemokine receptor expression confirms the identity of the sorted populations. Th1 cells are CCR6-CCR4- , Th2 cells are CCR6-CCR4+ , Th17 cells are CCR6+CCR4+ , and Treg cells show a mixed CCR6/CCR4 profile.

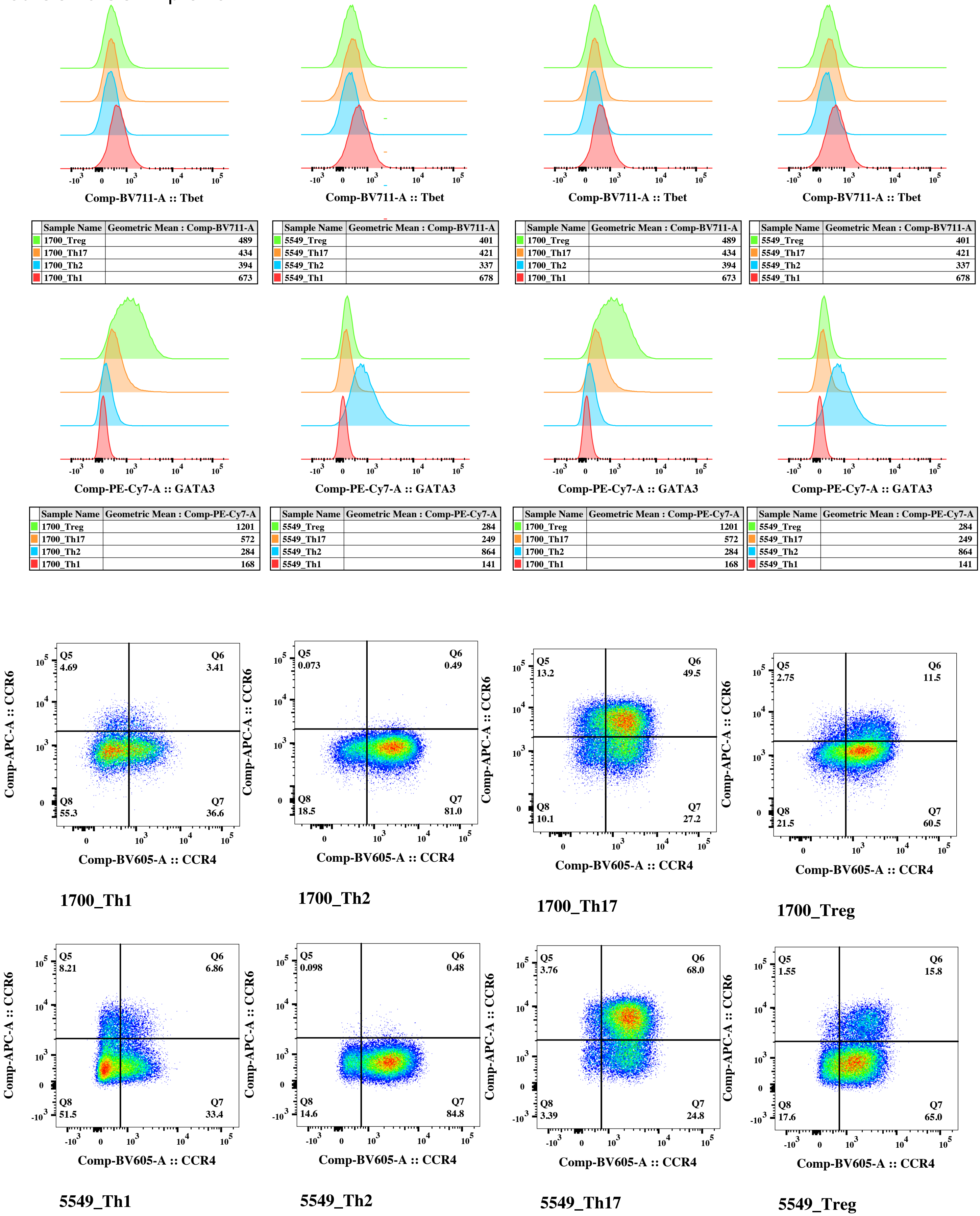

(B) Phenotypic characterization of day 14 in vitro expanded Th1, Th2, Th17, Tr1 & Treg subsets.
Top (Flow Plots): CD25 and FOXP3 expression is shown for Th1, Th2, Th17, Tr1, and Treg cells.
Middle (Dot Plots): These plots summarize the expanded populations, showing that FOXP3 expression is maintained at a high level only in Tregs. The "Average Fold" expansion is plotted for each subset. Chemokine receptor expression is also summarized, confirming Th1 cells as CXCR3+, Th2 cells as CCR4+CCR6neg, and Th17 cells as CCR4+CCR6+.
Bottom (Dot Plots): These plots compare Tr1 and Treg subsets, showing high expression of CD49b+LAG3+ in Tr1 cells and high expression of CTLA4+ and Helios+ in Treg cells.

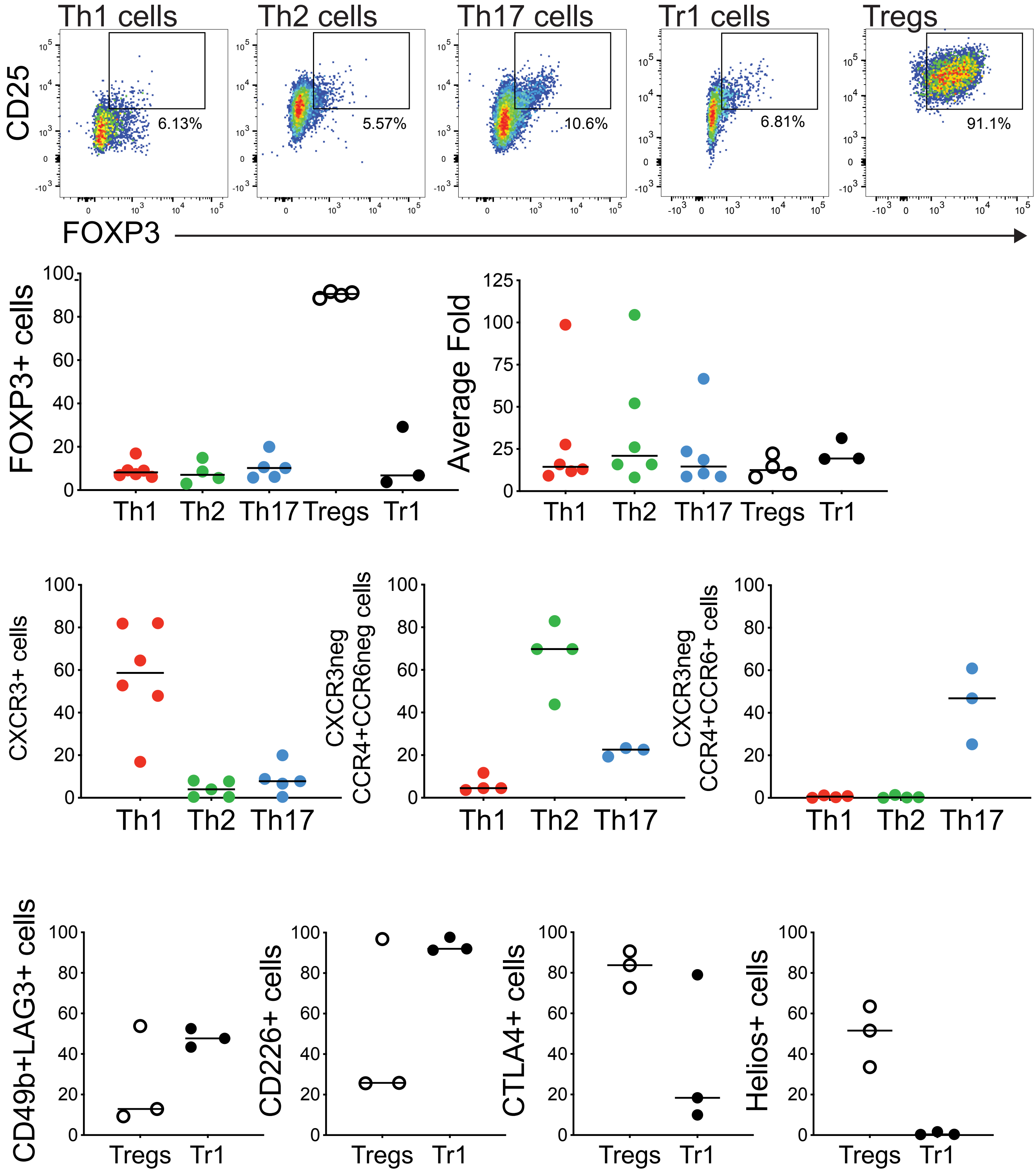

Figure S19 - Functional characterization of day 14 in vitro expanded Th1, Th2, and Th17 subsets.

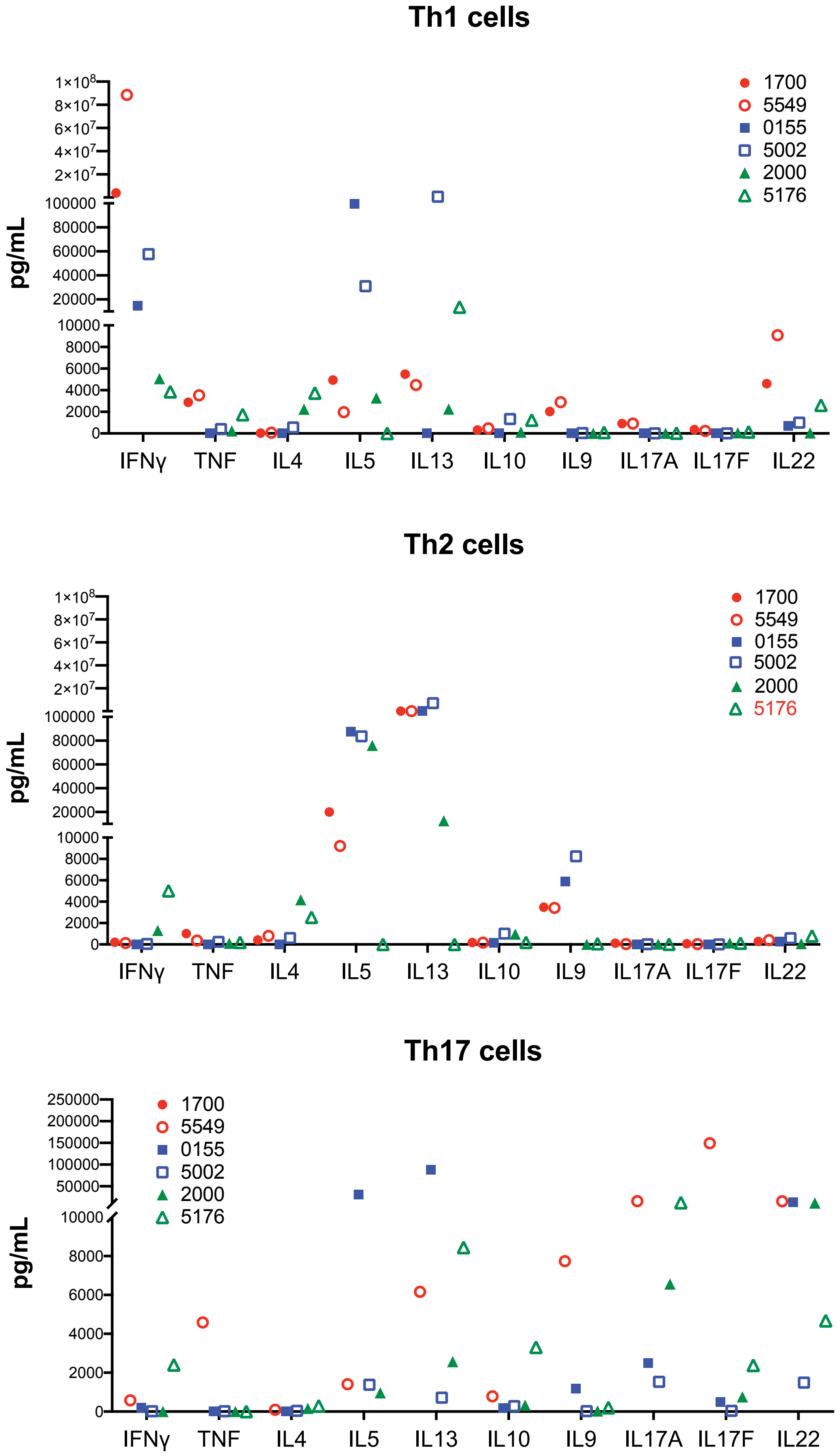

Supplement: Supplementary file 2 — Additional file 2. Supplementary Figures S1-S19.Figure S1. Full S-LDSC parameters across diverse cell types’ cREs annotation. Figure S2. Bar plots show number of significant heritability enrichment.Figure S3. Bar plots show number of significant conditional effect sizes. Figure S4. Intersections of V2G genes. Figure S5. Comparative predictive power of orthogonal V2G approaches. Figure S6. Cytokine/receptor gene enrichment across trait and cell type. Figure S7. Salmonella infection gene enrichment across trait and cell type. Figure S8. Sharing of V2G genes in enteroids across UC, CRO, and IBD. Figure S9. Gene ontology enrichment of cell type-specific V2G genes across cell type. Figure S10. Shared eGenes across different eQTL datasets with V2G. Figure S11. Shared eGenes across different eQTL datasets per locus. Figure S12. Proportion of eGenes identified by eQTL. Figure S13. Disrupted transcription factor binding motifs. Figure S14. Dot-plot shows effect sizes of SLE and RA variants on FDFT1 expression. Figure S15. Expression of BLK. Figure S16. Effect of lapaquistat on T cell activation. Figure S17. Gating strategies for Immune cells. Figure S18. Phenotypic characterization of the sorted Immune cells. Figure S19. Functional characterization of the expanded T-helper subsets. [file 13059_2025_3880_MOESM2_ESM.pdf]
